# Analysis of the Transcriptome of Blowfly *Chrysomya megacephala* (Fabricius) Larvae in Responses to Different Edible Oils

**DOI:** 10.1371/journal.pone.0063168

**Published:** 2013-05-14

**Authors:** Min Zhang, Hao Yu, Yanyan Yang, Chao Song, Xinjun Hu, Guren Zhang

**Affiliations:** State Key Laboratory for Biological Control/Institute of Entomology, Sun Yat-sen University, Guangzhou, Guangdong, China; Beijing Institute of Microbiology and Epidemiology, China

## Abstract

**Background:**

*Chrysomya megacephala* (Fabricius), a prevalent necrophagous blowfly that is easily mass reared, is noted for being a mechanical vector of pathogenic microorganisms, a pollinator of numerous crops, and a resource insect in forensic investigation in the postmortem interval. In the present study, in order to comprehensively understand the physiological and biochemical functions of *C. megacephala*, we performed RNA-sequencing and digital gene expression (DGE) profiling using Solexa/Illumina sequencing technology.

**Methodology/Principal Findings:**

A total of 39,098,662 clean reads were assembled into 27,588 unigenes with a mean length of 768 nt. All unigenes were searched against the Nt database, Nr database, Swiss-Prot, Cluster of Orthologous Groups (COG) and Kyoto Encyclopedia of Genes and Genome (KEGG) with the BLASTn or BLASTx algorithm (E-value<0.00001) for annotations. In total, 7,081 unigenes and 14,099 unigenes were functionally classified into 25 COG categories and 240 KEGG pathways, respectively. Furthermore, 20,216 unigenes were grouped into 48 sub-categories belonging to 3 main Gene Ontology (GO) categories (ontologies). Using the transcriptome data as references, we analyzed the differential gene expressions between a soybean oil-fed group (SOF) and a lard oil-fed group (LOF), compared to the negative control group (NC), using the DGE approach. We finally obtained 1,566 differentially expressed genes in SOF/NC, and 1,099 genes in LOF/NC. For further analysis, GO and KEGG functional enrichment were performed on all differentially expressed genes, and a group of differentially expressed candidate genes related to lipometabolism were identified.

**Conclusions/Significance:**

This study provides a global survey of *C. megacephal*a and provides the basis for further research on the functional genomics of this insect.

## Introduction

*Chrysomya megacephala* (oriental latrine fly), a member of Chrysomya (Insect, Diptera, Calliphoridae), is widely distributed in the United States, Australia, Argentina, Europe, and Asia [1]–[4]. It is famous for being used as a death investigator in forensic entomology [5]–[7] and as an economic insect pollinator in orchards [8]. In China, *C. megacephala* larvae (CML) have been used as traditional Chinese medicine for centuries to treat indigestion. It has been reported that insects can discriminate long-chain dietary fatty acids. *Drosophila melanogaster* (*D. melanogaster*) larvae prefer dietary unsaturated fatty acids while the adults generally prefer saturated fatty acids [9]. Low levels of diet fat can enhance the lifespan of the blowfly, while high-fat diets can cause more rapid death [10]. Diet also has a great impact on the fatty acid profiles of mosquitoes, which exhibit a high degree of dietary routing, characteristic of generalist feeders [11]. Actually, the fatty acids present in dietary lipids could directly affect the tissue phospholipid composition in *D. melanogaster* [12]. Like the mammalian counterpart, insect fatty acid desaturation is sensitive to dietary changes [13]–[14]. Recently, the suitability of CML as a protein source in animal feed [15] and as a potential alternative feedstock in biodiesel production has been explored [16]. However, molecular biology research on this blowfly species has been scarce. Therefore, the transcriptome and expression profiling data for this species are important for better understanding of the biological mechanisms of *C. megacephala*.

Whole transcriptome shotgun sequencing [17] (WTSS, also called RNA-seq), refers to the use of next-generation sequencing technologies (NGS, also called high-throughput sequencing or deep sequencing; Solexa/Illumina (Illumina), 454 (Roche) and SOLiD (ABI) are the typical NGS technologies [18]) to sequence cDNA in order to obtain information about the RNA content of the samples. Currently, Solexa/Illumina (Illumina) NGS is able to generate millions of DNA fragments simultaneously and provide Gigabases of data with high fidelity in a single machine running at a low cost compared to traditional methods [19]. Digital gene expression (DGE), an improved version of the serial analysis of gene expression (SAGE) technique, is a tag-based transcriptome sequencing approach for gene expression profiling analysis. RNA-seq has been used in transcriptome profiling studies for various applications in combination with the DGE technology, including the development model, regulatory mechanisms, and immune defense in various organisms [20]–[22].

In the present study, we obtained the transcriptome information of *C. megacephala* using RNA-seq (Solexa/Illumina), and analyzed the gene expression differences in the soybean oil-fed group (SOF) and the lard oil-fed group (LOF) compared to the negative control group (NC) of *C. megacephala* using DGE analysis. We obtained 27,588 assembled unigenes with 20,776 annotated to known databases,and identified a group of differentially expressed candidate genes related to lipometabolism in response to different edible oil. All these results provide a shortcut for identifying new functional genes and useful information for studying the molecular biology of the *C. megacephala*, especially the lipometabolism of *C. megacephala*, from which human obesity research may draw references.

## Materials and Methods

### Insects

The *C. megacephala* strain was loop-fed for more than 2 years in a 28°C incubator with RH65% in our laboratory. We fed three groups of *C. megacephala* from its egg stage in this study, the soybean oil-fed group (SOF), the lard oil-fed group (LOF), and the negative control group (NC). The feed of the soybean oil-fed group (lard oil-fed group) consisted of 800 g wheat bran, 200 g defatted fish meal, 250 g soybean oil (lard oil) and 1250 g water, while in the control group the 250 g oil was replaced by 250 g water. The egg inoculation amount was 0.80 g. Three replications were performed, and five three-day-old third instars larvae were collected as a sample for RNA extraction in each replication. The whole body (containing mid gut) was used for RNA extraction, and then three RNA from the replications were mixed with equal quality for RNA-sequencing.

### RNA extraction, library construction and RNA-sequencing

Total RNA was extracted from the negative control group using standard protocols (Trizol) and then treated with DNase to remove potential genomic DNA contamination according to the manufacturer's protocols. The RNA integrity (with a minimum RNA integrity number (RIN) value of 6.0) and concentration were evaluated using the Agilent 2100 Bioanalyzer (Agilent Technologies).

The sample for transcriptome analysis was prepared using Illumina's kit following the manufacturer's recommendations. In brief, beads with Oligo (dT) were used to isolate poly (A) mRNA after total RNA was collected from the negative control group. Fragmentation buffer was added to cut the mRNA into short fragments. Using these short fragments as templates, random hexamer-primer was used to synthesize the first-strand cDNA. The second-strand cDNA was synthesized in a mixture of buffer, dNTPs, RNaseH and DNA polymerase I. The short fragments were purified with QiaQuick PCR extraction kit and resolved with EB buffer for end reparation and adding poly (A). After that, the short fragments were connected with sequencing adapters. Following agarose gel electrophoresis, suitable fragments were selected to be templates for PCR amplification. In the final step, the library could be sequenced using Illumina HiSeq™ 2000.

### Analysis of the transcriptome results

Transforming the image data into sequence data with base calling, raw reads were produced from the sequencing machines. Then we filtered all the raw sequences to remove reads with adaptors, low quality sequences (reads with low quality nucleotides (sequencing quality value<10) more than 20%), and reads with unknown nucleotides of more than 5%. All clean read data has been deposited in the NIH Short Read Archive (SRA) database (Accession No. SRX206470).

Transcriptome *de novo* assembly was carried out with a short reads assembling program called Trinity [23]. Firstly, reads with a certain length of overlap were combined to form longer fragments, which were called contigs. Then the reads were mapped back to the contigs. It was able to detect contigs from the same transcript as well as the distances between these contigs from the paired-end reads. In the next step, the contigs were connected to obtain sequences that cannot be extended on either end, where the sequences were defined as unigenes. After clustering, the unigenes were divided into two classes. One was clusters with a prefix of CL, and the other was singletons with a prefix of unigene. In the final step, BLASTx alignment (e-value<0.00001) between unigenes and protein databases such as nr, Swiss-Prot, KEGG and COG was performed, and the best aligning results were used to decide the sequence direction of the unigenes. If the results of the different databases conflicted with each other, a priority order of nr, Swiss-Prot, KEGG and COG should be followed. When a unigene happened to be aligned to none of the above databases, a software named ESTScan [24] would be used to decide the sequence direction. Except for the alignment between the unigenes and the above protein databases (e-value<0.00001), we also aligned the unigenes to nucleotide databases using BLASTn (Nt, e-value<0.00001) to obtain the protein functional annotations with the highest sequence similarity to the given unigenes in all the databases.

With nr annotation, we used Blast2GO program [25] to obtain the GO (http://www.geneontology.org/) annotations of the unigenes. We also used WEGO software [26] to conduct GO functional classification for all unigenes. The COG and KEGG [27] pathways annotations were performed using BLAST software against the COG database and the KEGG database.

### Digital gene expression (DGE) library construction and sequencing

Tag library construction for the three groups was performed in parallel using Illumina Gene Expression Sample Prep Kit and Solexa Sequencing Chip (flowcell). Briefly, mRNA was purified from 6 µg total RNA using Oligo (dT) magnetic beads, and then the double-strand cDNAs were directly synthesized on the magnetic beads using the Oligo (dT) primer. The bead-bound cDNAs were subsequently digested with restriction enzyme *NlaIII*, which specifically recognized and cut off the CATG sites. The fragments apart from the 3′ cDNA fragments connected to Oligo (dT) beads were washed away and the Illumina adaptor 1 was ligated to the sticky 5′ end of the digested bead-bound cDNA fragments. The junction of the Illumina adaptor 1 and CATG site was the recognition site of *MmeI*, which cut at 17 bp downstream of the CATG site, producing tags with adaptor 1. After removing the 3′ fragments with magnetic beads precipitation, the Illumina adaptor 2 was ligated to the 3′ ends of tags, acquiring tags with different adaptors at both ends to form a tag library. The library was amplified using linear PCR for 15 cycles, and 105 bp fragments were purified using 6% TBE PAGE gel electrophoresis. After denaturation, the single-chain molecules were attached to the Illumina Sequencing Chip (flow cell) for sequencing. Each tunnel generated millions of raw reads with a sequencing length of 49 bp. The data sets are available at the NIH SRA database with the accession number: SRX206472.

### Mapping DGE tags to the reference transcriptome database

Prior to mapping reads to the reference transcriptome database, we filtered all sequences to remove the adaptor sequence, low quality sequences (tags with unknown sequences ‘N’), empty tags (sequence with only adaptor sequences but no tags), tags which are too long or too short (leaving the tags of 21 nt), and tags with a copy number of 1 (probably sequencing error). For annotation, all tags were mapped to our transcriptome reference database and only 1 nucleotide mismatch was considered. All the clean tags mapped to reference sequences from multiple genes were filtered and the remaining tags were designed as unambiguous tags. For gene expression analysis, the number of expressed tags was calculated and then normalized to TPM (number of transcripts per million tags) [28]–[29], and the differentially expressed tags were used for mapping and annotation.

### Identification of differentially expressed genes

A statistical analysis of the frequency of each tag in the different cDNA libraries was performed to compare gene expression in SOF, LOF and NC. Statistical comparison was performed with custom written scripts using the method described by Audic *et al.* [30]. We used “FDR≤0.001 and the absolute value of log2Ratio ≥1” as the threshold to judge the significance of the gene expression difference. At last, the differentially expressed genes were aligned to the GO database for GO functional enrichment analysis (the correct threshold of the hypergeometric test≤0.05), we also mapped all differentially expressed genes to the KEGG database and looked for significantly enriched KEGG pathways (the correct threshold of the hypergeometric test≤0.05) compared to the transcriptome database.

### Experimental Validation

#### Reverse transcription PCR (RT-PCR) confirmation

To confirm the non-redundant transcripts, the cDNA of 6 annotated non-redundant transcripts was selected randomly and amplified using RT-PCR. Total RNA was extracted from NC with the previous protocols and then the cDNA was synthesized according to the manufacturer's protocol (PrimeScript^RT^ reagent Kit, TaKaRa, Japan). The PCR (*Premix tag* Version2.0, TaKaRa, Japan) was performed using ABI Veriti™96 Well Thermal Cycler under a 3 Step PCR program: 36 cycles of 94°C for 30 sec, 60°C for 30 sec and 72°C for 30 sec. The target fragments in the products of the RT-PCR amplification were purified from the gel following the manufacturer's specifications of the Universal DNA Purification Kit (TIANGEN, Peking, China), and then the target fragments were cloned into the pMD 18-T vector (TaKaRa, Japan) and amplified using the *E.coli* Competent Cells DH5α for Sanger sequencing (Invitrogen Biotechnology Co.Ltd., Shanghai, China). The forward and reverse primers used for the RT-PCR are shown in Table S1. All the sequenced target fragments were aligned to the non-redundant transcripts in RNA-Seq using BLASTn (e-value<0.00001).

#### Real-time quantitative PCR (qRT-PCR) confirmation

To confirm the DGE results, we designed 6 pairs (Table S1) of primer to perform qRT-PCR analysis on 5 up-regulated genes and 1 down-regulated gene (used for RT-PCR). Total RNA was extracted independently from biological replicates as described for the DGE experiments. The first-strand cDNA was synthesized according to the protocols described above. The qRT-PCR was performed using a Roche480 Real-Time PCR system (Roche) with SYBR-Green detection (SYBR *Premix Ex Taq*, TaKaRa, Japan) according to the manufacturer's instructions. The qRT-PCR program was: 40 cycles of 95°C for 5 sec, 60°C for 20 sec and 72°C for 20 sec. Each reaction was run in triplicate, after which the average threshold cycle (Ct) was calculated per sample. We also cloned the beta-actin gene (Genbank Accession No.: KC207081) of *C. megacephala* as an internal gene to normalize the expression levels. The relative expression of genes was calculated using the2^−ΔΔCt^ method [31]. All the qRT-PCR products were cloned into *E.coli* Competent Cells DH5α for Sanger sequencing.

## Results

### Illumina sequencing and de novo assembly

**Figure 1:**
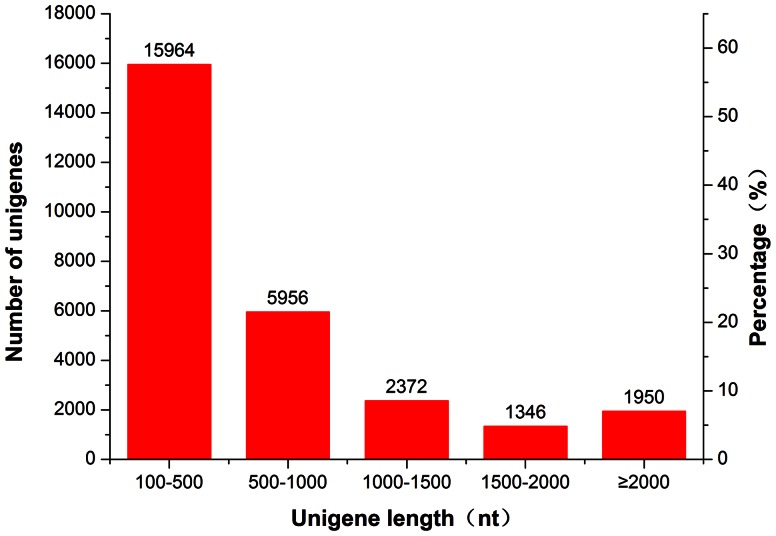
Unigene size distribution. The left y-axis indicates the number of unigenes to different unigene length scales, and the right y-axis indicates their percentages.

A total of 39,098,662 clean reads with a cumulative length of 3,518,879,580 nt were generated by Illumina sequencing and assembled into 60,793 contigs where the <100 bp ones were excluded (Table 1). Using Trinity software by the manner of paired-end reads, the contigs were further assembled into 27,588 unigenes containing a length of more than 200 bp, including 716 clusters and 26,872 singletons with a mean length of 768 bp. The size distribution indicated that the ratio of unigenes with a length of 200–1000 bp was approximately 80%, while the lengths of 5,668 (20.55%) unigenes were more than 1000 bp (Fig. 1). These results demonstrated that the size of the contigs and unigenes was significantly larger than in previous insect transcriptome projects [32]–[36], which might be because our project used novel software with an advanced algorithm.

**Table 1:** Summary for the Illumina sequencing and *de novo* assembly.

	Clean reads	Contigs	Unigenes
**Number of sequences**	39,098,662	60,793	27,588
**Total Length (bp)**	3,518,879,580	21,194,858	21,194,858
**Mean Length (bp)**	90	361	768
**N50(bp)**	-	677	1,252

N50 = median length of all non-redundant consensus sequences.

### Annotation of unigenes

In order to annotate the unigenes, all unigenes were searched against the nr database, Swiss-Prot, Cluster of Orthologous Groups (COG), Kyoto Encyclopedia of Genes and Genome (KEGG) and nt database with the BLASTx or BLASTn algorithm (E-value<0.00001). A total of 20,776 (75.27% of all unigenes) unigenes were annotated (Table 2), where the nr database (20,216 unigenes were annotated, 73.28%) had the largest match, followed by the Swiss-Prot (16,068, 58.24%) and KEGG (14,099, 51.11%) databases. And the rest (6,812, 24.73%) not annotated to the existing databases were the potential sources of novel genes.

**Table 2:** Summary for annotation of unigenes (E-value<0.00001).

Database	Number of Unigene-annotated	Percentage
**nr**	20,216	73.28%
**Swiss-Prot**	16,068	58.24%
**KEGG**	14,099	51.11%
**COG**	7,081	25.67%
**nt**	10,789	39.11%
**All Unigene-annotated**	20,776	75.27%
**All Unigene**	27,588	100%

### Gene Ontology (GO) and clusters of orthologous groups (COG) classification

**Figure 2:**
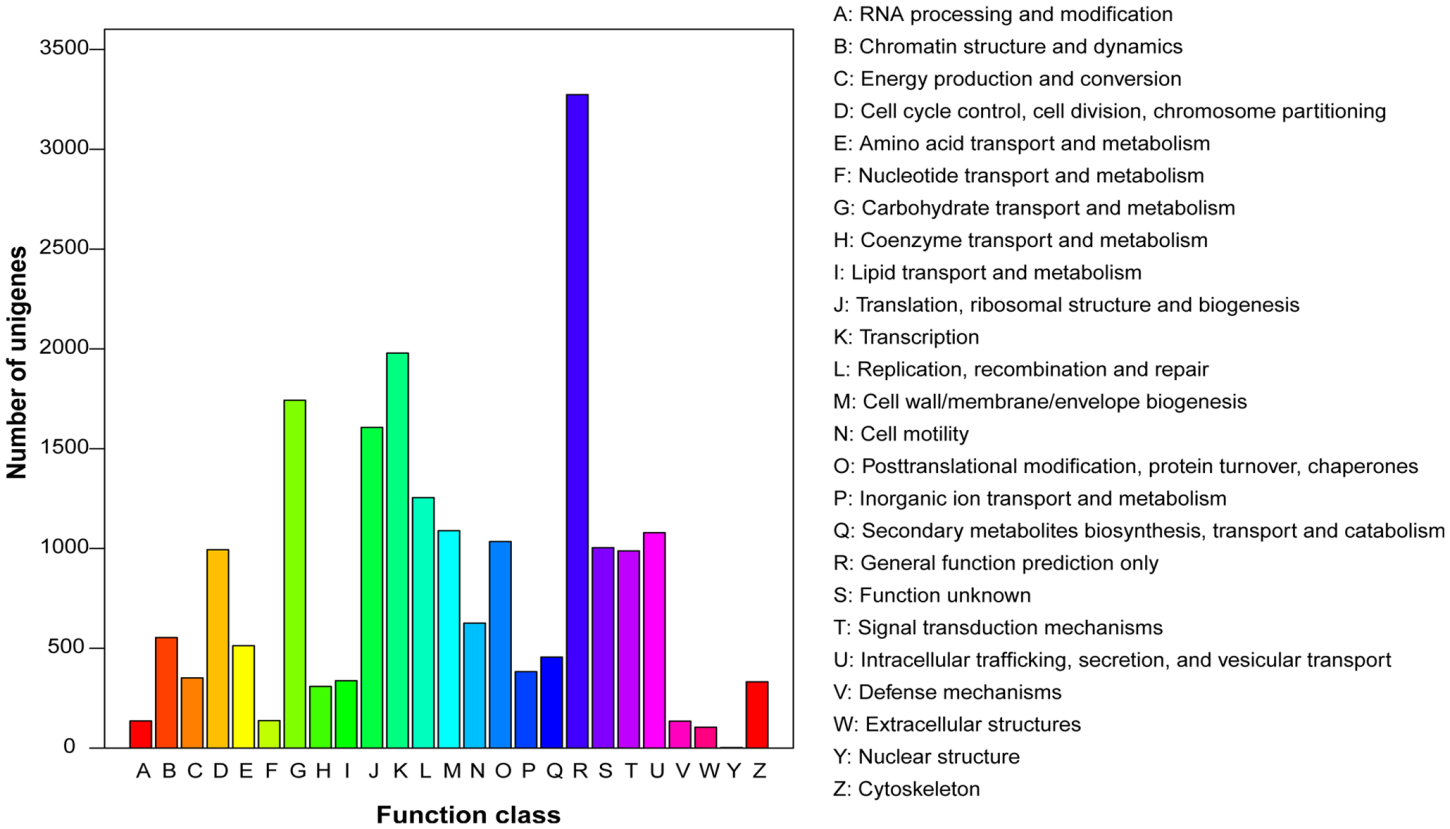
Histogram presentation of GO classification of Unigenes. 20,216 unigenes were grouped into 48 sub-categories, which were divided into three categories: biological processes, cellular components, and molecular functions.

GO assignments were used to classify the functions of the predicted *C. megacephala* genes. Using the Blast2GO and WEGO softwares, 20,216 unigenes annotated to the nr database previously were grouped into 48 sub-categories belonging to three main GO categories (biological process, cellular component and molecular function) (Fig. 2). It was found that the top 6 sub-categories were ‘Cell (2,798 unigenes, 13.84% of unigenes annotated to the nr database)’, ‘Cellular process (2,573, 12.73%)’, ‘Cell part (2,493, 12.33%)’, ‘Binding (2,109, 10.43%)’, ‘Metabolic process (1,958, 9.69%)’ and ‘Catalytic activity (1,816, 8.98%)’. Coincidentally, the top 6 sub-categories belonged to the three main GO categories averagely. In each of the three main categories, ‘Cellular process (2,573 unigenes, 12.73%)’, ‘Cell (2,798 unigenes, 13.84%)’ and ‘Binding (2,109 unigenes, 10.43%)’ terms were dominant, respectively.

**Figure 3:**
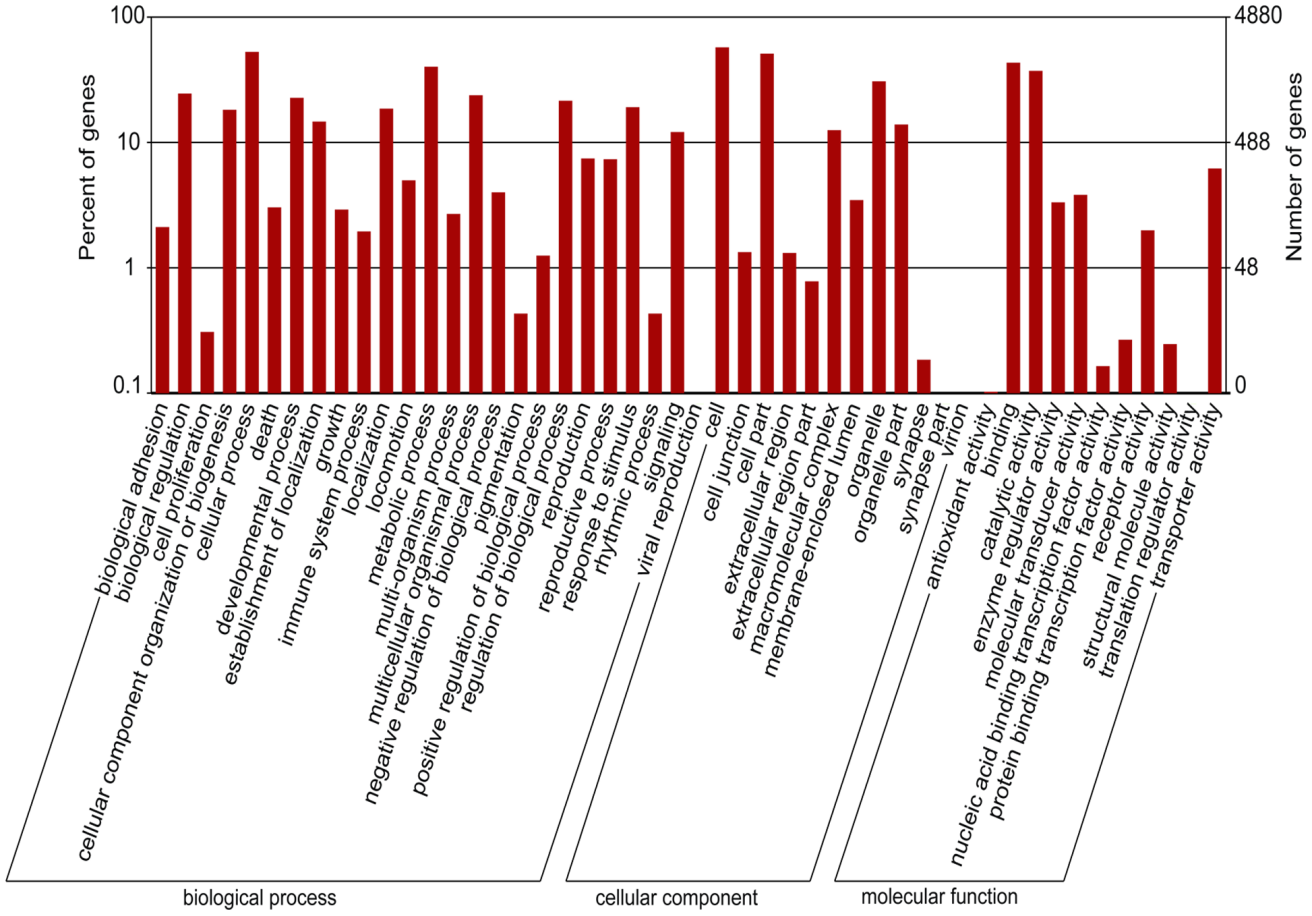
Histogram presentation of COG function classification of Unigenes. 7,081 unigenes were classified functionally into 25 COG categories.

We also used COG classifications to further evaluate the completeness of our transcriptome library and the effectiveness of our annotation process. In total, out of 27,588 unigenes, 7,081 unigenes were classified functionally into 25 COG categories (Fig. 3). The cluster for ‘General function prediction only (3,274 unigenes, 46.24% of unigenes annotated to COG database)’ represented the largest group, and the following groups were ‘Transcription (1,979, 27.95%)’, ‘Carbohydrate transport and metabolism translation (1,743, 24.62%)’ and ‘Translation, ribosomal structure and biogenesis (1,607, 22.69%)’.

KEGG is a database capable of analyzing gene products during the metabolic process and the related gene function in the cellular processes. To identify the biological pathways that are active in *C. megacephala*, we mapped all the unigenes to the reference canonical pathways in KEGG. A total of 14,099 unigenes were assigned to 240 KEGG pathways (Table S2), where ‘Metabolic pathways (1,797 unigenes, 12.75%)’ is dominant, followed by ‘Pathways in cancer (552, 3.92%)’ and ‘Focal adhesion (501, 3.55%)’. All the 15 pathways related to lipid metabolism in the third instar larvae of *C. megacephala* are listed in Table 3. ‘Glycerophospholipid metabolism’, ‘Glycerolipid metabolism’ and ‘Fatty acid metabolism’ were the top 3 pathways related to lipometabolism.

**Table 3:** The KEGG pathways related to lipid metabolism in the third instar larvae of *C. megacephala*.

#	Pathway	Unigene	Percentage	Pathway ID
1	Glycerophospholipid metabolism	153	1.09%	ko00564
2	Glycerolipid metabolism	111	0.79%	ko00561
3	Fatty acid metabolism	84	0.60%	ko00071
4	alpha-Linolenic acid metabolism	74	0.52%	ko00592
5	Ether lipid metabolism	44	0.31%	ko00565
6	Sphingolipid metabolism	44	0.31%	ko00600
7	Biosynthesis of unsaturated fatty acids	42	0.30%	ko01040
8	Steroid hormone biosynthesis	40	0.28%	ko00140
9	Arachidonic acid metabolism	26	0.18%	ko00590
10	Fatty acid biosynthesis	22	0.16%	ko00061
11	Linoleic acid metabolism	22	0.16%	ko00591
12	Steroid biosynthesis	20	0.14%	ko00100
13	Synthesis and degradation of ketone bodies	15	0.11%	ko00072
14	Fatty acid elongation	11	0.08%	ko00062
15	Primary bile acid biosynthesis	8	0.06%	ko00120

### Digital gene expression (DGE) library sequencing

To investigate the responses of *C. megacephala* to different edible oils, we investigated the global gene expression profiles in the third instar larvae using the Solexa/Illumina's DGE system. Three *C. megacephala* larvae DGE libraries were sequenced: the negative control group (NC), the lard-fed group (LOF) and the soybean oil-fed group (SOF). The major characteristics of these three libraries are summarized in Table 4. We finally obtained 3.39 million (98.25% of the total raw tags), 3.42 million (97.57%) and 3.64 million (97.98%) clean tags in each library after filtering the adaptor tags, low quality tags and tags of copy number<2. The NC library had the lowest number of both distinct tags and tags. In each library, the highly expressed genes (copy number>100) showed percentages of greater than 75% among the clean tags, but their distribution of distinct clean tags did not exceed 7%. In contrast, the genes with a low expression level (copy number<10) showed a broad distribution of distinct clean tags. The data showed that only a small proportion of genes had a high expression level. Saturation analysis of the capacity of libraries showed that the library capacity (increment of distinct tags) had approached saturation when the number of sequencing tags was large enough (Fig. S1).

**Table 4:** Major characteristics of DGE libraries and tag mapping to the *C. megacephala* transcriptome database.

Summary	Negative control group				Soya-bean oil-fed group				Lard-fed group			
	Distinct Tag	Percentage	Total Tag	Percentage	Distinct Tag	Percentage	Total Tag	Percentage	Distinct Tag	Percentage	Total Tag	Percentage
**Raw Data**	109,620	100%	3,454,222	100%	162,742	100%	3,504,620	100%	144,532	100%	3,711,506	100%
Low Quality Tag	293	0.27%	384	0.01%	354	0.22%	412	0.01%	333	0.23%	431	0.01%
Adaptor Tag	114	0.10%	145	0.00%	395	0.24%	534	0.02%	85	0.06%	105	0.00%
Tag CopyNum<2	60,014	54.75%	60,014	1.74%	84,376	51.85%	84,376	2.41%	74,610	51.62%	74,610	2.01%
Clean Tag	49,199	44.88%	3,393,679	98.25%	77,617	47.69%	3,419,298	97.57%	69,504	48.09%	3,636,360	97.98%
CopyNum≥2	49,199	100%	3,393,679	100%	77,617	100%	3,419,298	100%	69,504	100%	3,636,360	100%
CopyNum>5	22,609	45.95%	3,317,220	97.75%	34,318	44.21%	3,293,763	96.33%	31,657	45.55%	3,526,962	96.99%
CopyNum>10	15,310	31.12%	3,261,666	96.11%	22,116	28.49%	3,200,847	93.61%	20,702	29.79%	3,443,671	94.70%
CopyNum>20	9,937	20.20%	3,182,970	93.79%	13,685	17.63%	3,077,253	90.00%	12,830	18.46%	3,328,556	91.54%
CopyNum>50	5,147	10.46%	3,029,301	89.26%	6,622	8.53%	2,853,569	83.45%	6,417	9.23%	3,125,441	85.95%
CopyNum>100	2,970	6.04%	2,875,351	84.73%	3,680	4.74%	2,647,369	77.42%	3,588	5.16%	2,926,900	80.49%
**Tag Mapping**												
Mapping to Unigene	25,884	52.61%	2,846,574	83.88%	46,112	59.41%	2,775,689	81.18%	40,675	58.52%	3,009,257	82.75%
Mapping to Genome	1,388	2.82%	42,306	1.24%	1,498	1.93%	41,355	1.21%	1,471	2.12%	44,901	1.23%
Unknown Tag	21,927	44.57%	504,799	14.87%	30,007	38.66%	602,254	17.61%	27,358	39.36%	582,202	16.01%
Unambiguous Mapping	22,645	46.03%	2,510,148	73.97%	40,219	51.82%	2,463,371	72.04%	35,659	51.30%	2,666,067	73.32%
**All Tag-mapped Genes**	-	-	9,680	35.09%	-	-	12,396	44.93%	-	-	11,673	42.31%
Unambiguous Tag-mapped Genes	-	-	7,864	28.51%	-	-	10,363	37.56%	-	-	9,696	35.15%

Mapping to Unigene represents the number of all tags mapped to the *C. megacephala* transcriptome database, Mapping to Genome represents the number of all tags mapped to the *Drosophila melanogaster* genome database, Unambiguous Mapping represents the number of unambiguous tags mapped to the *C. megacephala* transcriptome database, and unambiguous tags/genes indicate the tags matched only to one gene. All Tag-mapped Genes represents the number of all genes (27,588 unigenes, sense & antisense) with a mapped tag at least, Unambiguous Tag-mapped Genes represents the number of genes (unigenes, sense & antisense) matched only to one kind of tag.

### Analysis of tag mapping

When we mapped the tags to the reference transcriptome database, we retrieved all CATG+17 tags in a gene (unigene) as the gene's reference tags, and 53,857 reference tags with 47,497 (88.19%) unambiguous reference tags were finally obtained (Table 4). All the reference tags were distributed in 18,682 (67.72%) unigenes. Considering polymorphism across samples, tolerances were set to allow one mismatch in each alignment. Most tags matched to the 1^st^ or 2^nd^ 3′ CATG site in the reference transcripts (Fig. S2). Among the clean tags, the number of sequences that could be mapped to the *C. megacephala* transcriptome database ranged from 2.78 to 3.01 million, and its percentage was from 81.18 to 83.88% in the three libraries. Correspondingly, 52.61–59.41% of distinct clean tags mapped to the unigenes, 46.03–51.82% of the distinct clean tags mapped unambiguously to the unigenes, and 38.66–44.57% of the distinct clean tags did not map to the *C. megacephala* transcriptome database. The occurrence of unknown tags was probably due to the absence of *C. megacephala* genome sequencing.

Moreover, the NC library had the lowest number of both tag-mapped genes (9,680, 35.09%) and unambiguous tag-mapped genes (7,864, 28.51%), indicating that more transcripts were expressed in the other two libraries than the NC library.

Bidirectional transcription is an important regulation of gene expression. Sequencing tags mapped to the complementary strand of the sense gene suggests that its antisense strand also has transcripts, and this gene may use the sense-antisense regulation. The DGE profiling of the mouse showed that 51% of the detectable unigenes contained bidirectional transcription [29]. We found evidence for bidirectional transcription in 3,321 to 4,945 (Table S3) unigenes in each library, in which the percentage was approximately 45% of the detectable unigenes. In addition, antisense-strand specific transcripts were from 3,889 to 4,043 (27%). We also found that the antisense transcripts were expressed at substantial levels in *C. megacephala*. The ratio of sense to antisense strand of the transcripts was approximately 1.0 for all libraries. This suggests the transcriptional regulation in *C. megacephala* acts equally on the sense and antisense strand.

### Identification of differentially expressed genes among the different treatments

To gain the global transcriptional changes in the different oil-fed groups, we applied the method described previously [30] to identify the differentially expressed genes by pairwise comparisons among the three different treatments. In our analysis, the differentially expressed gene was defined as false discovery rate (FDR) [37] <0.001 and estimated absolute log2-fold change >1. We also listed the GO and nr annotations of the differentially expressed genes. The complete lists of differentially expressed genes are shown in Table S4.

**Figure 4:**
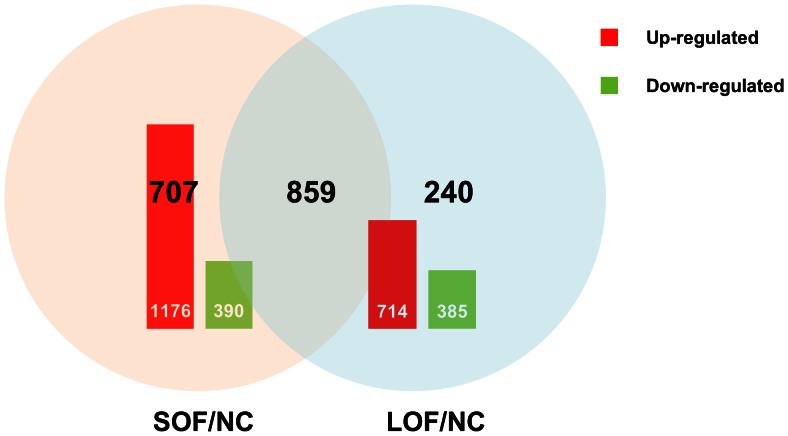
Changes in gene expression profiling among the different treatments. Up-(red) and down-regulated (green) unigenes were quantified and presented by histogram, comparisons of differentially expressed genes in SOF/NC and LOF/NC presented by Venn chart.

The results revealed 1,566 differentially expressed genes with 1176 up-regulated genes and 390 down-regulated ones in the soybean oil-fed group compared to the control group library (SOF/NC) (Fig. 4). The comparison between the lard oil-fed group and the control group library also revealed significant variations in expression. A total of 1,099 genes, including 714 up-regulated and 385 down-regulated genes, were identified in the lard oil-fed group compared to the control group library (LOF/NC). There were 859 differentially expressed genes belonging to both SOF/NC and LOF/NC. Unexpectedly, most of the differentially expressed genes demonstrated coordinated regulation both in SOF/NC and LOF/NC, and only 2 differentially expressed genes (unigene 9469, unigene 161) performed antagonistic regulation in the two comparisons. Of the differentially expressed genes in SOF/NC and LOF/NC, 190 and 140 genes could not be annotated, respectively; namely, their functions are unknown. We also performed comparison between the soybean oil-fed group and lard oil-fed group libraries (SOF/LOF). In total, 250 genes included 80 up-regulated genes and 170 down-regulated genes demonstrated significant changes.

### Gene Ontology functional enrichment analysis for DGEs

To understand the functions of these differentially expressed genes, all the differentially expressed genes were mapped to the GO database and compared to the whole transcriptome background (Table S5). All the differentially expressed genes in LOF/NC can be categorized into 1,058 GO terms (134 cellular component terms, 208 molecular function terms, and 716 biological process terms), and the differentially expressed genes in SOF/NC can be categorized into 1,348 GO terms (162 cellular component terms, 239 molecular function terms, and 947 biological process terms). The terms related to lipid metabolism, which belonged to molecular function and biological process categories only, are summarized in Table S6. 35 and 52 differentially expressed genes related to lipid metabolism in LOF/NC and SOF/NC were obtained, respectively. All the differentially expressed genes in SOF/LOF can be categorized into 426 GO terms (47 cellular component terms, 67 molecular function terms, and 312 biological process terms). Unexpectedly, we did not find that the differentially expressed genes were related to lipid metabolism in SOF/LOF.

### Pathway enrichment analysis for DGEs

Different genes usually cooperate with each other to exercise their biological functions. Pathway-based analysis helps to further understand the biological functions of the genes. To characterize the functional consequences of gene expression changes associated with different oil treatments, we performed pathway analysis of differentially expressed genes based on the KEGG database with p-value of <0.05 as the threshold.

As shown in Table S7, of the 218 pathways, 11 pathways demonstrated significant changes in the soybean oil-fed group compared to the control group library, while the pathways related to lipid metabolism was ‘Pancreatic secretion’. The comparison between the lard oil-fed group and the control group library also revealed 16 significant pathways out of 207 pathways demonstrated significant changes, and the pathways related to lipid metabolism included ‘Fat digestion and absorption’, ‘Glycerolipid metabolism’ and ‘Pancreatic secretion’. In addition, 13 pathways demonstrated significant changes in SOF/LOF, and the pathways related to lipid metabolism were ‘Steroid biosynthesis’ and ‘Pancreatic secretion’, where ‘Pancreatic secretion’ also demonstrated significant changes in SOF/NC and LOF/NC.

### Experimental validation

#### Validation of transcriptome data by RT-PCR

We selected 1 down-regulated gene (crystalline alpha B (CRYAB, Unigene 10603), and 5 up-regulated genes (radixin (RDX, Unigene 11981), xanthine dehydrogenase (XDH, Unigene 18353), scavenger receptor protein (SCAR, Unigene 3030), signal recognition particle subunit (SRP72, Unigene 11020), and cathepsin B mRNA 3′-untranslated-region-binding protein (CBBP, Unigene 11922) for RT-PCR conformation. In this analysis, all 6 primer pairs led to a band with the expected size and the identities of PCR products were confirmed by Sanger sequencing. The results are listed in Table S1. The exact identities between the RT-PCR fragments and non-redundant transcripts from RNA-Seq (BLASTn, e-value<0.00001) indicated the reliability of our transcriptome data.

#### Validation of DGE data by qRT-PCR

**Figure 5:**
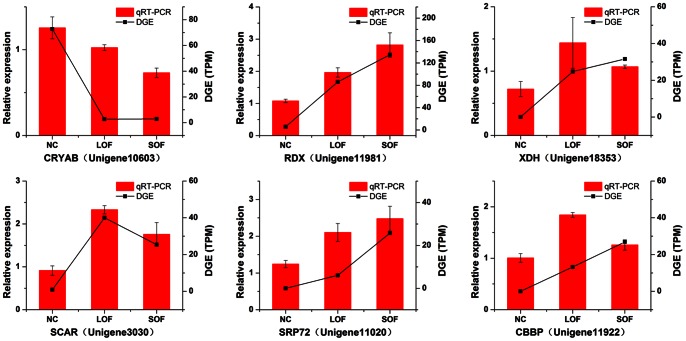
qRT-PCR validation of DGE results. The left y-axis indicates the relative expression level obtained by qRT-PCR (2^−ΔΔCt^), which were presented as fold changes in gene expression normalized to the *actin* gene in each group, and the right y-axis indicates the TPM (transcripts per million mapped reads) obtained by DGE.

To confirm the differential DGE results by Solexa/Illumina sequencing, we also performed qRT-PCR (Fig. 5) for the genes validated above. Data were presented as fold changes in gene expression normalized to the *actin* gene and relative to the NC sample. The qRT-PCR results were identical to the direction of change obtained by DGE analysis. This correlation indicated the reliability of the DGE results.

## Discussion

Although NGS technologies have been developed for the rapid sequencing and characterization of many transcriptomes, the sequence data of *C. megacephala* was scarce until recently. Obtaining more sequence information was a priority for deciphering gene function in *C. megacephala*. In the present study,we applied transcriptome profiling to study the responses of this blowfly to different oils, which is the first study for obtaining whole transcriptome information in this species. We sequenced the *C. megacephala* transcriptome using Solexa/Illumina NGS, and obtained more than 3.5 billion clean base sequences. The clean base sequences were finally assembled into 27,588 unigenes with a satisfactory mean length. Our results provide the most extensive published sequencing resource for *C. megacephala*.

A total of 20,776 (75.27% of all unigenes) unigenes has been annotated to the known databases (Nr, Swiss-Prot, COG, KEGG and Nt) for a comprehensive function analysis, and the rest of the unigenes (6,812, 24.73%) not annotated to the existing databases would be potential sources of novel genes. In insects, lipids stored in droplets, which consist of a triglyceride core surrounded by a layer of phospholipids and embedded proteins, represent the major component of the fat body and the main source of metabolic fuel [38]. In our KEGG pathway enrichment analysis, ‘Glycerophospholipid metabolism’ and ‘Glycerolipid metabolism’ were the most dominant pathways (Table 3) related to lipid metabolism in *C. megacephala*. Compared to the 16 reference pathways related to animal lipid metabolism in KEGG (http://www.genome.jp/kegg/pathway.html#lipid), ‘Secondary bile acid biosynthesis’ was not found. Interestingly, 843 unigenes could be classified into ‘Pathways in cancer’. It has been reported that many genes of *Amphimedon queenslandica* were also implicated in cancer, suggesting the remote origin of cancer and oncogenes [39] [40]. The ‘Pathways in cancer’ may provide supports for the theme. These annotations provide a valuable resource for investigating specific processes, functions and pathways in *C. megacephala*.

Insects and mammals, as well as some other organisms, employ several identical mechanisms of deposition and mobilization of triglycerides. Hence, the application of insect models to the investigation of the basic questions related to lipid storage and mobilization will be an important reference [38]. In insects, a change in the fatty acid content can affect female fecundity [41] [10] and remating [42], as well as their production of sex pheromones [43]. In addition, the lipid was the primary fuel during metamorphosis, which accounted for >80% of the total metabolism [44]. The presence and composition of fatty acids in cell membranes can affect cold adaptation in *Drosophila* [45], polyunsaturated fatty acids can also activate the *Drosophila* light-sensitive channels [46]. Through DGE analysis, we obtained 1,566 differentially expressed genes in SOF/NC, and 1,099 genes in LOF/NC, where the differentially expressed genes related to lipid metabolism could be categorized into some ancestry GO terms, such as lipid metabolic process, lipid localization and fatty acid ligase activity. They provide important reference information for further study on the functions and mechanisms of lipometabolism.

In summary, we obtained the whole transcriptome sequences of the *C. megacephala* by high-throughput sequencing, and analyzed the gene expression differences in the soybean oil-fed group and the lard oil-fed group compared to the negative control group. As more data will be obtained from other species, our data in this study will be further annotated and analyzed. The results will provide a solid basis for research on the molecular mechanisms of the biological traits of *C. megacephala*.

### Conclusions

Our transcriptome results provide a comprehensive sequence resource for future *C. megacephala* study, establishing an important public information platform for functional genomic studies in *C. megacephala*. Furthermore, the DGE data provide comprehensive gene expression information for the CML, which will facilitate our understanding of the lipometabolism mechanisms of *C. megacephala*.

## Supporting Information

Figure S1 **Library size on gene identify ratio.** The left y-axis indicates the percentage of genes identified, and the right y-axis indicates the total tag number. The figure showed that the library capacity (increment of distinct tags) has approached saturation when the number of sequencing tags reach 2 million.
(TIF)

Figure S2 **Tag position and gene expression.** The left y-axis indicates the total tag number, and the right y-axis indicates the N-th tag in gene from the 3′ end. The figure showed that most tags matched to the 1st or 2nd 3′ CATG site in the reference transcripts.
(TIF)

Table S1 **The primers and BLAST results of experiment validation.**
(XLS)

Table S2 **The KEGG pathways of all unigenes.**
(XLSX)

Table S3 **Antisense gene expression.**
(XLSX)

Table S4 **Differentially expressed genes.**
(XLS)

Table S5 **GO analysis of differential expressed genes.**
(XLSX)

Table S6 **The differentially expressed genes related to lipid metabolism.**
(XLSX)

Table S7 **Pathway analysis of differentially expressed genes.**
(XLS)
