# Supplementary figures and images for: Analysis of the Transcriptome of Blowfly Chrysomya megacephala (Fabricius) Larvae in Responses to Different Edible Oils
Source: PLoS One. 2013 May 14;8(5):e63168. doi: 10.1371/journal.pone.0063168 (PMC3653882; doi:10.1371/journal.pone.0063168)

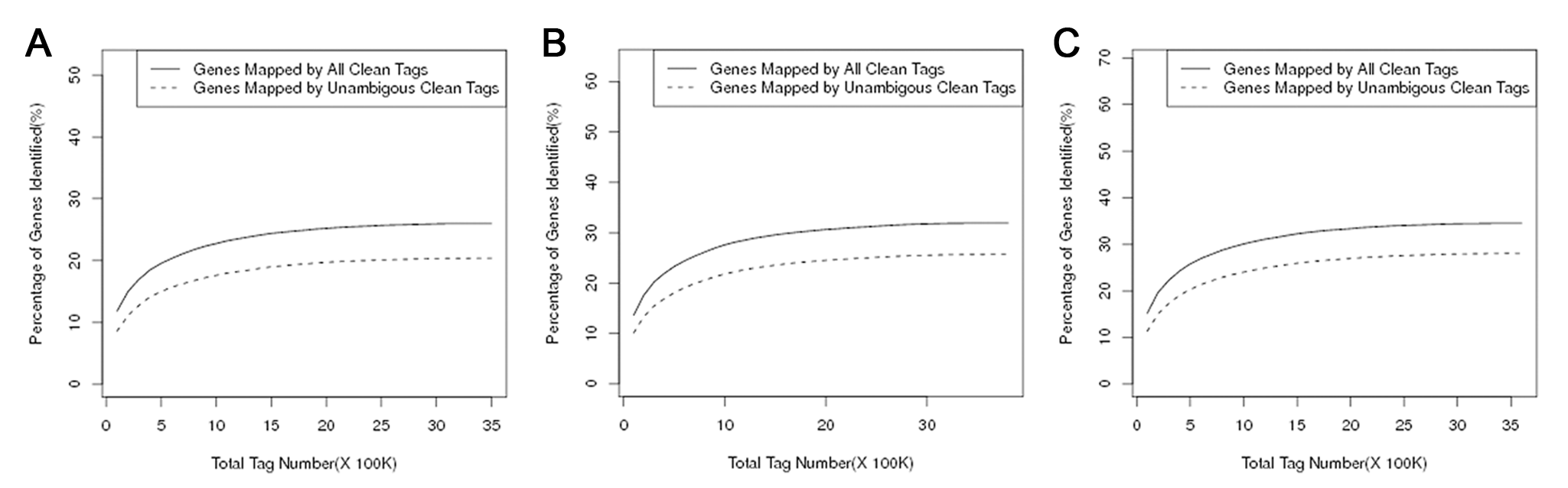

Supplement: Figure S1 — Library size on gene identify ratio. The left y-axis indicates the percentage of genes identified, and the right y-axis indicates the total tag number. The figure showed that the library capacity (increment of distinct tags) has approached saturation when the number of sequencing tags reach 2 million. (TIF) [file pone.0063168.s001.tif]

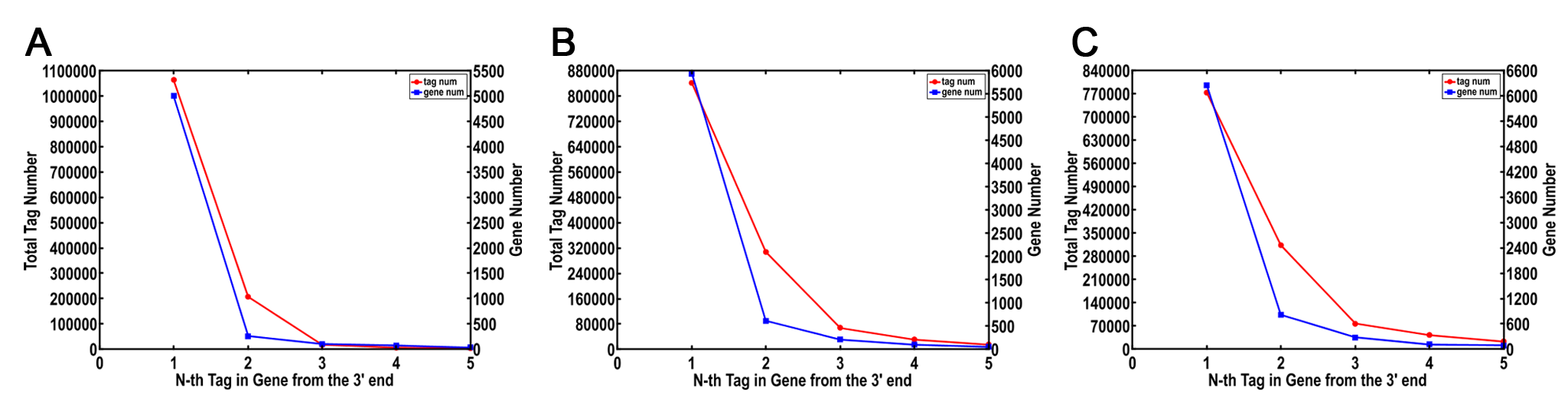

Supplement: Figure S2 — Tag position and gene expression. The left y-axis indicates the total tag number, and the right y-axis indicates the N-th tag in gene from the 3′ end. The figure showed that most tags matched to the 1st or 2nd 3′ CATG site in the reference transcripts. (TIF) [file pone.0063168.s002.tif]
